# Interhospital Transfer Dependency and Delays to Arterial Puncture in UK Thrombectomy Centres: A National Observational Study

**DOI:** 10.7759/cureus.117306

**Published:** 2026-10-01

**Authors:** Marwa Bibi

**Affiliations:** 1 Emergency Medicine, Royal London Hospital, London, GBR

**Keywords:** acute ischaemic stroke (ais), arterial puncture, door-in-door-out (dido) time, interhospital transfer, large vessel occlusion (lvo), mechanical thrombectomy (mt), reperfusion time

## Abstract

Introduction

Mechanical thrombectomy (MT) is a highly time-dependent intervention, with functional outcomes deteriorating as the interval from stroke onset to arterial puncture lengthens. Interhospital transfer pathways broaden regional access to MT but introduce additional steps that may prolong this interval. A widely held assumption in health service planning is that hubs treating a greater proportion of transferred patients therefore experience greater average delay; however, this assumption is yet to be examined at a national level. This study evaluates the association between transfer dependency and delay to arterial puncture at a national level and identifies the operational contributors to delay within the transferred pathway.

Materials and methods

This was a two-year retrospective observational analysis of aggregated, centre-level data from the Sentinel Stroke National Audit Programme (SSNAP) for all MT procedures nationally between April 2023 and March 2025. Direct and transferred pathways were compared for each independent hyperacute stroke unit (HASU), and transfer-associated delay (TAD) was derived in this study to examine the association between transfer dependency and delay. Transferred pathways were decomposed into door-in-door-out (DIDO), conveyance, and receiving-centre door-to-puncture (DTP) intervals to identify operational contributors to delay. Pearson and Spearman correlations were calculated, and sensitivity analyses (small-caseload exclusion, volume weighting, absolute transferred volume and a negative control) were performed to assess robustness.

Results

Among 7,203 MT procedures (3,421 in 2023/24 and 3,782 in 2024/25), approximately 60% followed interhospital transfer. The dependency-TAD association was inconsistent between years (r = +0.50, 95% CI 0.10 to 0.76, p = 0.017 in 2023/24 versus r = -0.06, 95% CI -0.46 to 0.36, p = 0.78 in 2024/25) and supports a cautious interpretation of the relationship between transfer dependency and delay. Structural decomposition of transferred pathways demonstrated meaningful associations between DIDO, DTP and TAD: median DIDO was 131 and 129 minutes respectively in the two years and accounted for 55-57% of the median total transferred interval. Centre-level transfer dependency, DIDO and DTP values were highly stable year-on-year (r = 0.86-0.89), suggesting that these delays are consistent and reproducible features of pathway organisation. Both DTP and DIDO correlated with TAD substantially in both years.

Conclusion

Whilst transferred pathways demonstrated substantial additional delay to arterial puncture, higher transfer dependency was not consistently associated with delay. DIDO was uniformly prolonged across the national network and, together with receiving-centre DTP, correlated with TAD in both years, suggesting that delay within the transferred pathway is more closely related to organisational factors. Both represent potentially valuable targets for regional quality improvement initiatives.

## Introduction

Background

Mechanical thrombectomy (MT) is the standard of care for acute ischaemic stroke caused by large-vessel occlusion, with treatment benefit that declines steeply as the interval from onset to arterial puncture lengthens [1-2]. International guidelines therefore emphasise minimising every component of pre-hospital and in-hospital delay [3-4]. Because MT is delivered at a limited number of specialist centres [5], a large proportion of eligible patients first present to a nearer non-thrombectomy hospital before transfer to a thrombectomy hub, often referred to as the ‘drip-and-ship’ pathway.

Despite major advances in thrombectomy capability and the expansion of 'drip-and-ship' networks, timely access to treatment remains a significant challenge within transferred pathways [4]. In the STRATIS registry, transferred patients experienced approximately one hour of additional system delay compared with direct arrivals, with measurable consequences for functional outcome [6]. Comparable penalties have been reported in Dutch and other European cohorts [7], and meta-analytic comparison of mothership and drip-and-ship models confirms a consistent time cost of transfer to arterial puncture in the region of 45-60 minutes [8-10].

A widely invoked assumption in health services is that thrombectomy centres handling a high proportion of transferred patients are subject to ‘capacity strain’, whereby transfer-heavy hubs experience greater transfer-associated delay (TAD) because their processes are saturated by inbound transfers [11-12]. This assumption is intuitively appealing, features in systems-of-care and consensus discussions of hub capacity and transfer demand [12], and is sometimes used to argue for demand diversion or additional hub capacity. Despite its influence, the assumption has not been tested at national scale. The present study tests this assumption using national, real-world audit data within the UK stroke system.

In the United Kingdom, the drip-and-ship model is the dominant pathway for MT [13]. All thrombectomy procedures are captured by the Sentinel Stroke National Audit Programme (SSNAP), a nationally mandated audit of stroke care in England, Wales and Northern Ireland [13-14]. Given the scope of this audit, these data offer a unique opportunity to ascertain whether transfer dependency is associated with additional delay to arterial puncture at a national level, and to identify which operational factors contribute to delay.

Objectives

The primary objective of this study was to evaluate the centre-level relationship between transfer dependency and time to arterial puncture. The secondary objective was to identify operational bottlenecks within the transferred pathway that could contribute to delay.

## Materials and methods

Study design and data source

This retrospective national observational study used publicly available, aggregate, anonymised SSNAP data downloaded from its Comprehensive Knowledge Archive Network (CKAN) open-data directory [14]. Service-published definitions and reporting methodology were used for all variables extracted [13,15]. This study was reported in accordance with the Strengthening the Reporting of Observational Studies in Epidemiology (STROBE) statement [16] and the REporting of studies Conducted using Observational Routinely collected health Data (RECORD) statement [17].

Setting and sample selection

The study included MT procedures reported over the 24-month period from April 2023 to March 2025 [14]. Of 26 centres reporting MT activity, exclusions were applied to centres whose pathway structure precluded estimation of TAD (Figure 1), leaving 22 centres in 2023/24 and 23 in 2024/25.

**Figure 1:**
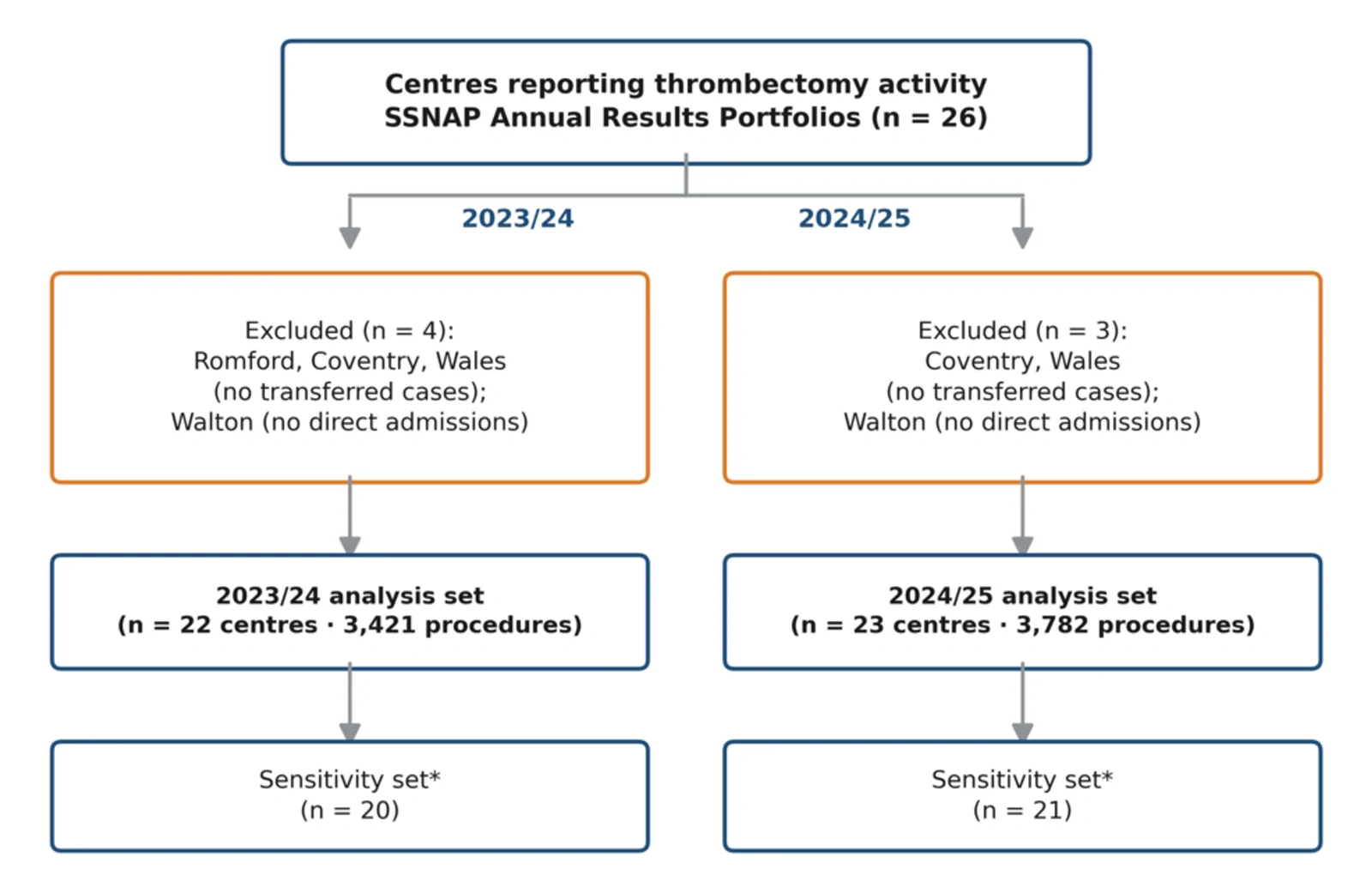
Centre selection and analysis sets Centres were excluded where transfer-associated delay (TAD) could not be estimated. SSNAP = Sentinel Stroke National Audit Programme; Romford = Queens Hospital Romford; Coventry = University Hospital Coventry; Wales = University Hospital of Wales; Walton = Walton Centre. *Sensitivity sets exclude centres with fewer than 10 transferred cases.

Two centres had very small transferred caseloads (James Cook University Hospital and University College London Hospitals, n = 6 each in 2024/25); both were excluded in sensitivity analyses, and their inclusion did not materially influence any results.

Outcome definitions

Transfer dependency is the percentage of a centre’s total MT caseload arriving from interhospital transfer. Direct-admission puncture (DAP) interval refers to the median duration from arrival to arterial puncture in patients admitted directly to a hyperacute stroke unit (HASU).

Transferred-pathway puncture (TPP) interval is the median duration from arrival at the first referring hospital to arterial puncture at the receiving HASU. TAD is a study-derived metric (rather than a routinely validated performance measure) calculated as the difference between each HASU's median TPP and DAP intervals, and utilised in this study to quantify the additional system delay attributable to the transfer pathway, consistent with direct-versus-transferred contrasts in prior registry studies [6-7,18].

The transferred pathway was decomposed into three sequential components, each measured independently at centre level as a median: 1) Door-in-door-out (DIDO) is time from arrival at the first referring hospital until physical discharge from that hospital [19-21]; 2) Conveyance is time from discharge from the first referring hospital to formal arrival at the receiving HASU, and 3) Receiving-centre door-to-puncture (DTP) is time from arrival at the receiving HASU until arterial puncture.

All intervals are centre-level medians as published in the portfolio; no individual-level data were accessed. DIDO was contextualised against the proposed 60-minute benchmark for primary stroke centres [19-20], which recent national data from other systems suggest is rarely achieved [21].

Statistical analysis

Median values with interquartile ranges (IQR) were used for descriptive comparisons across centres, with national SSNAP aggregates used as a comparator. Centre-level Pearson correlation coefficients (r) and Spearman rank correlation coefficients (ρ) were calculated to examine the association between transfer dependency and workflow measures, with two-tailed p-values and 95% confidence intervals for r derived using the Fisher z-transformation.

Pre-specified sensitivity analyses comprised: (i) exclusion of the two centres with fewer than 10 transferred cases (n = 20 in 2023/24; n = 21 in 2024/25); (ii) volume-weighted Pearson correlation, weighting each centre by its transferred caseload; (iii) correlation of absolute transferred volume (rather than proportional dependency) with TAD; (iv) correlation of dependency with the transferred interval alone; and (v) a negative-control correlation of dependency with the direct-admission interval. Two-tailed p-values for Spearman correlations were derived by permutation. 

Descriptive tabulation was performed in Microsoft Excel 365, Version 2608 (Microsoft Corporation, Redmond, WA, USA); correlation coefficients, confidence intervals and sensitivity analyses were computed in Python 3.11 with SciPy 1.x (Python Software Foundation, Wilmington, DE, USA). 

## Results

Network activity and transfer profile

Transferred patients accounted for a median of 61.4% (range 2.9-75.4) of MT activity in 2023/24 and 61.7% (range 7.4-80.3) in 2024/25 (Figure 2). In 2024/25, the centre median referral-to-puncture interval for transferred patients was 234 min (range 188-343), compared with 145 min (range 96-198) for direct arrivals, yielding a median TAD of 98 min (range 37-198); the corresponding 2023/24 values were 243 min transferred, 126 min direct and a median TAD of 104.5 min (range 43-199).

**Figure 2:**
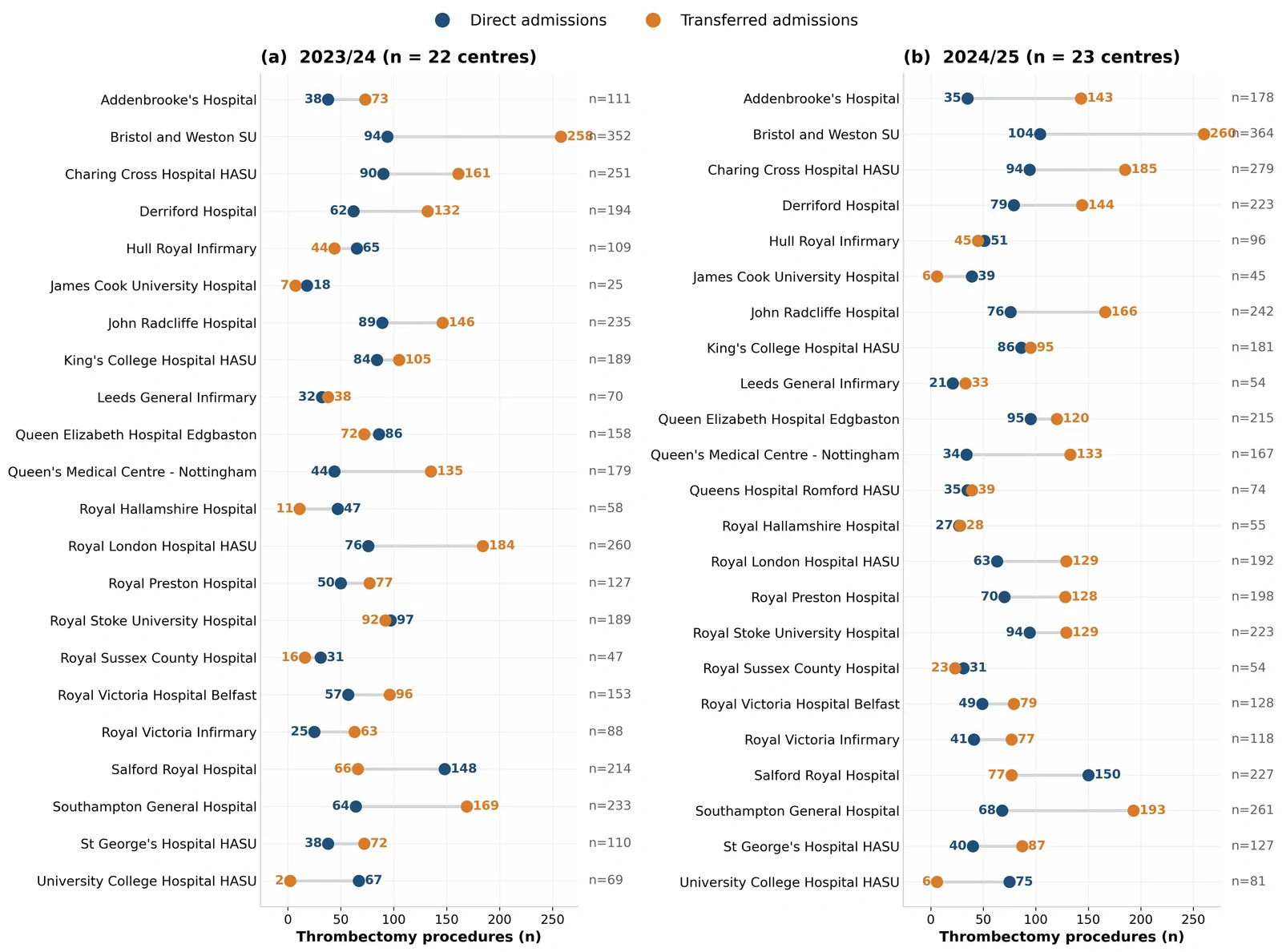
Network activity and transfer profile of centres over two years Direct (blue) and transferred (orange) mechanical thrombectomy procedures per centre in (a) April 2023 to March 2024 and (b) April 2024 to March 2025. Connecting lines show the within-centre split between direct and transferred arrivals; n is the centre's total thrombectomy caseload. Queen's Hospital Romford performed no transferred procedures in 2023/24 and was excluded from that year's analysis set. HASU = hyperacute stroke unit.

Within the transferred pathway, medians in 2023/24 and 2024/25 respectively were: DIDO 131 min (range 70-202) and 129 min (range 82-203); conveyance 46 min (30-76) and 46 min (24-124); receiving-centre DTP 42.5 min (10-100) and 47 min (14-113). DIDO accounted for a median of 56.9% and 55.1% of the transferred interval in the two years, and pre-arrival components (DIDO plus conveyance) for 78.5% and 74.8%. Fifteen of 22 centres in 2023/24 and 16 of 23 in 2024/25 had a DIDO of at least 120 min - twice the proposed 60-minute benchmark [19-20]. This is among the most important operational observations of this study and reinforces the potential value of improvement initiatives targeting the referring-hospital phase.

Primary analysis: transfer dependency and TAD

Although transferred pathways were consistently slower than direct pathways, the magnitude of this delay was not proportional to transfer dependency at centre level, and the dependency-TAD association was inconsistent between years (Figure 3). 

**Figure 3:**
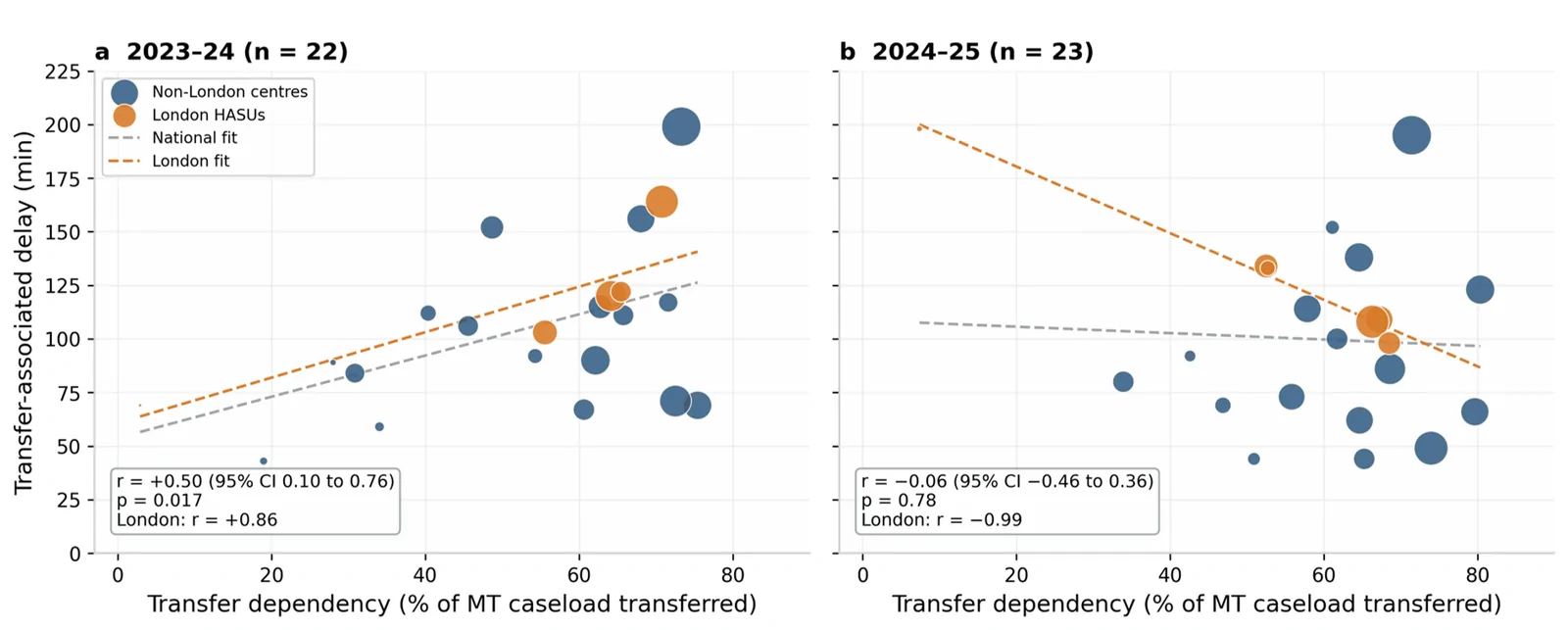
Transfer dependency versus transfer-associated delay (TAD) in two consecutive audit years (a) 2023/24 (n = 22): moderate positive association (r = +0.50, p = 0.017). (b) 2024/25 (n = 23): no association (r = −0.06, p = 0.78). Bubble area is proportional to absolute transferred caseload; London hyperacute stroke units are highlighted in orange. In a post-hoc subgroup analysis, the London correlation was r = +0.86 (n = 5, p = 0.06) in 2023/24 and r = −0.99 (n = 6, p < 0.001) in 2024/25, while non-London correlations were non-significant in both years (n = 17; r = 0.42, p = 0.09 and r = 0.35, p = 0.17); these small subgroups are presented descriptively. HASU = hyperacute stroke unit; MT = mechanical thrombectomy.

In 2023/24, dependency correlated moderately and significantly with TAD (Pearson r = +0.50, 95% CI 0.10 to 0.76, p = 0.017; Spearman ρ = 0.49, permutation p = 0.021). The positive association persisted after exclusion of centres with fewer than 10 transferred cases (n = 20; r = 0.47, 95% CI 0.04 to 0.76, p = 0.036) and was attenuated by volume weighting (r = 0.30).

In 2024/25, there was no association (Pearson r = -0.06, 95% CI -0.46 to 0.36, p = 0.78; Spearman ρ = 0.03, permutation p = 0.90); the point estimate was effectively zero, and the confidence interval excluded strong correlations (|r| greater than ~0.5). All sensitivity analyses were consistent with the primary result: exclusion of the two centres with fewer than 10 transferred cases (n = 21; r = 0.12, 95% CI -0.33 to 0.53, p = 0.60), volume weighting (r = 0.08), absolute transferred volume (r = 0.16, p = 0.48), dependency against the transferred interval alone (r = -0.06, p = 0.79), and the negative control against the direct-admission interval (r = 0.003, p = 0.99).

Secondary analysis: structural decomposition of the transferred pathway

Decomposition of the transferred pathway indicates that centres experienced substantial delays before interhospital conveyance had even begun and that this delay was consistent between years (Table 1).

**Table 1:** Structural pathway decomposition and correlation analyses MT = mechanical thrombectomy; TAD = transfer-associated delay; DIDO = door-in-door-out time; DTP = door-to-puncture time; HASU = hyperacute stroke unit. Year-on-year correlations are across the 22 centres estimable in both years (dependency, DIDO and DTP p < 0.001; TAD p = 0.026; conveyance p = 0.13).

Measure	2023/24 (n = 22)	2024/25 (n = 23)	Year-on-year r
MT procedures, n (% transferred)	3,421 (59.0)	3,782 (61.5)	-
Transfer dependency, median % (range)	61.4 (2.9-75.4)	61.7 (7.4-80.3)	0.89
TAD, median min (range)	104.5 (43-199)	98 (37-198)	0.47
DIDO, median min (range)	131 (70-202)	129 (82-203)	0.86
Centres with DIDO ≥120 min, n/N	15/22	16/23	-
DIDO share of transferred interval, median %	56.9	55.1	-
Pre-arrival share (DIDO + conveyance), median %	78.5	74.8	-
Conveyance, median min (range)	46 (30-76)	46 (24-124)	0.34
Receiving-centre DTP, median min (range)	42.5 (10-100)	47 (14-113)	0.88
Dependency vs TAD, r (95% CI; p)	+0.50 (0.10 to 0.76; 0.017)	-0.06 (-0.46 to 0.36; 0.78)	-
DIDO vs TAD, r (p)	0.66 (0.001)	0.54 (0.008)	-
Receiving DTP vs TAD, r (p)	0.45 (0.035)	0.52 (0.012)	-
Conveyance vs TAD, r (p)	0.48 (0.023)	not significant	-

Referring-hospital DIDO correlated with TAD in 2023/24 (r = 0.66, 95% CI 0.33 to 0.85, p = 0.001) and 2024/25 (r = 0.54, 95% CI 0.16 to 0.78, p = 0.008), as did receiving-centre DTP (r = 0.45, 95% CI 0.04 to 0.73, p = 0.035; and r = 0.52, 95% CI 0.13 to 0.77, p = 0.012) (Figure 4). Conveyance time correlated with TAD in 2023/24 (r = 0.48, 95% CI 0.07 to 0.75, p = 0.023) but not in 2024/25.

**Figure 4:**
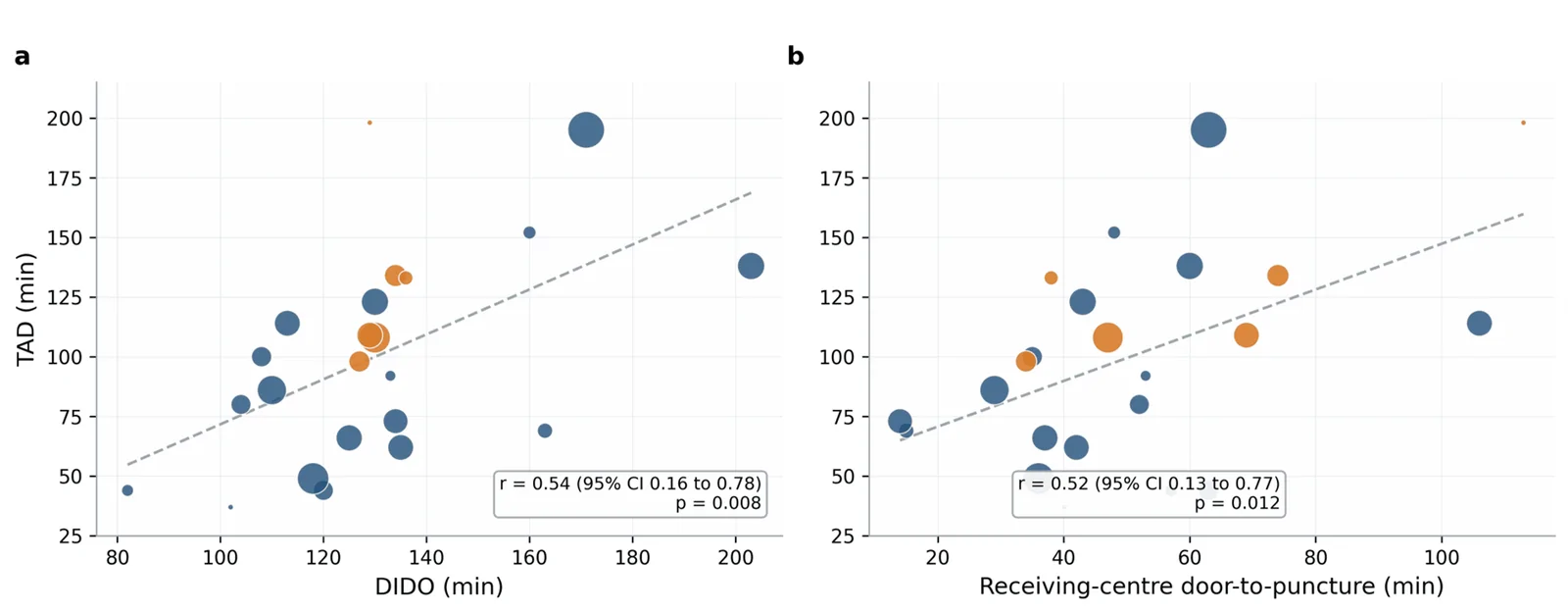
Transfer-associated delay versus DIDO and DTP, 2024/25 (n = 23) (a) Referring-hospital door-in-door-out time versus TAD (r = 0.54, 95% CI 0.16–0.78, p = 0.008). (b) Receiving-centre door-to-puncture time versus TAD (r = 0.52, 95% CI 0.13–0.77, p = 0.012). Each point represents one thrombectomy centre (n = 23); corresponding 2023/24 correlations were r = 0.66 and r = 0.45. TAD = transfer-associated delay; DIDO = door-in-door-out time; DTP = door-to-puncture time.

## Discussion

Principal findings

This analysis reveals two findings for stroke system design. First, interhospital transfer was associated with a substantial and consistent time penalty to arterial puncture across the national thrombectomy network. This finding is consistent with previous studies demonstrating that secondary transfer introduces additional delays when compared with direct presentation to thrombectomy-capable centres [7,10]. Second, and contrary to hypothesis, there was no consistent relationship between a thrombectomy centre’s dependency on transferred patients and the additional delay those patients experienced. The capacity-strain assumption that transfer-heavy hubs suffer systematically greater TAD is therefore not consistently supported. 

The 2024/25 null is consistent with the volume-outcome literature, in which higher-volume endovascular centres achieve faster, not slower, treatment [22-26], and with evidence that referring-centre volume does not predict the speed of transfer processes [27]. High-volume centres may develop standardised transfer acceptance protocols, parallel workflow and protected angiography access that offset their greater inbound demand - mechanisms consistently recommended in systems-of-care and national consensus guidance [11-12] and shown to shorten transfer and reperfusion times at both referring and receiving centres [28-30].

Therefore, routing decisions should remain sensitive to local geography and system performance rather than to assumptions about hub strain, and should continue to incorporate multiple operational and clinical factors beyond transfer dependency alone.

Pathway decomposition and operational delays

Findings from this analysis suggest that delays within transferred thrombectomy pathways are driven predominantly by processes occurring before arrival at the thrombectomy centre, particularly within the referring hospital phase. Across the national network, DIDO remained prolonged at all centres analysed and no service approached the recommended 60-minute operational target [19-20]. These findings align with a growing literature identifying the door-in-door-out period as the dominant and most modifiable segment of the drip-and-ship pathway [19-21], suggesting that the prolonged intervals observed here represent an appropriate target for pathway optimisation related to imaging acquisition, ambulance activation, referral and departure processes [13,18].

However, transfer delay cannot be attributed solely to the pre-arrival phase. Although DIDO was the strongest correlate of TAD in both years, its prolongation was near-universal, and receiving-centre DTP exhibited marked between-centre variation and was likewise associated with TAD in both years. Previous descriptions of hub-and-spoke thrombectomy pathways have similarly identified receiving-centre workflow and coordination as important determinants of overall treatment time [6,20], and this remains a plausible target for service improvement.

Implications for service improvement and stroke system design

The relative uniformity of prolonged DIDO across centres suggests that shared systemic factors, rather than isolated centre-specific inefficiencies, influence pre-transfer phase delay. Delays to specialist referral, local diagnostic image acquisition, and fragmented ambulance dispatch may represent common, network-wide targets for service improvement [4,28].

Receiving-centre processing also matters. Whereas DIDO was uniformly prolonged, receiving-centre DTP varied markedly between centres, indicating that some hubs consistently process transferred patients faster than others. Operational adjustments (early team activation, direct-to-angiography pathways and suite availability) have been shown to reduce receiving-centre times [29-30] and could be adopted in such centres to reduce delay.

These national data, whilst ecological, identify the same leverage points at system level in two consecutive years and strengthen the case for standardisation of the pre-transfer phase and optimisation of receiving-centre DTP across transfer pathways. Implementation of these strategies will nonetheless require adaptation to local service organisation, geography and resource availability. 

Limitations

Principal limitations include the ecological design: all analyses were conducted with centre-level medians, and associations observed at centre level cannot be assumed to hold for individual patients. Centre-level medians also reduce statistical precision so the magnitude of the reported correlations should be interpreted cautiously. Residual confounding is likely and cannot be addressed: no case-mix variables (age, stroke severity, onset-to-arrival times), comorbidity or imaging-selection data were available, and it is unclear whether transferred and directly admitted patients differ systematically in ways that may influence pathway performance. The TAD metric is study-derived rather than a routinely validated performance measure. The analysis covers two audit years with slightly different centre sets (n = 22 and n = 23), and the year-to-year change in the primary association, while itself informative, limits causal interpretation of either year's estimate. Finally, no outcome data were available, so delay cannot be related to functional recovery.

## Conclusions

This study shows that interhospital transfer was associated with a substantial and consistent time penalty to arterial puncture across the UK national thrombectomy network, but the magnitude of this delay was not proportional to transfer dependency. DIDO represented a substantial and uniformly prolonged component of the transferred pathway and is the most consistent, network-wide target for quality improvement identified in this analysis. Receiving-centre DTP additionally showed an independent association with TAD in both years and marked between-centre variation, identifying this as a secondary, centre-specific target for improvement.

Future prospective, patient-level evaluation linking SSNAP data with case-mix adjustment and functional outcomes would be valuable to test whether the centre-level patterns reported here hold at patient level and whether DIDO and DTP improvements translate into better recovery. Process-mapping studies within high-DIDO networks would help identify the specific workflow steps amenable to standardisation.
